# Di­chlorido­diphenyl­bis­(thio­urea-κ*S*)tin(IV)

**DOI:** 10.1107/S1600536813024343

**Published:** 2013-09-12

**Authors:** Yaya Sow, Libasse Diop, Kieran C. Molloy, Gabriele Kociok-Köhn

**Affiliations:** a Laboratoire de Chimie Minerale et Analytique (LACHIMIA), Departement de Chimie, Faculte des Sciences et Techniques, Universite Cheikh Anta Diop, Dakar, Senegal; b Department of Chemistry, University of Bath, Bath BA2 7AY, England

## Abstract

The title compound, [Sn(C_6_H_5_)_2_Cl_2_(CH_4_N_2_S)_2_], has been obtained from the reaction between Sn(C_6_H_5_)_2_Cl_2_ and SC(NH_2_)_2_. The asymmetric unit consists of one half of the mol­ecular unit, the remainder generated by a twofold rotation axis located along the Cl—Sn—Cl bonds. The Sn^IV^ atom is coordinated by two phenyl groups, two Cl atoms and two thio­urea ligands in an all *trans* octa­hedral C_2_Cl_2_S_2_ environment. Individual mol­ecules are connected through N—H⋯Cl hydrogen bonds, leading to a three-dimensional network structure. Intra­molecular N—H⋯Cl hydrogen bonds are also present.

## Related literature

For background to organotin(IV) chemistry, see: Kapoor *et al.* (2005 ▶); Sadiq-ur-Rehman *et al.* (2007) ▶; Zhang *et al.* (2006 ▶). For organotin(IV) compounds exhibiting biological activity, see: Nath *et al.* (2001 ▶); Pellerito & Nagy (2002 ▶). For chlorido­tin(IV) complexes, see: Amini *et al.* (2002 ▶); Müller *et al.* (2008 ▶). For tin(IV) complexes containing thio­urea groups, see: Donaldson *et al.* (1984 ▶); Sow *et al.* (2012 ▶); Wirth *et al.* (1998 ▶).
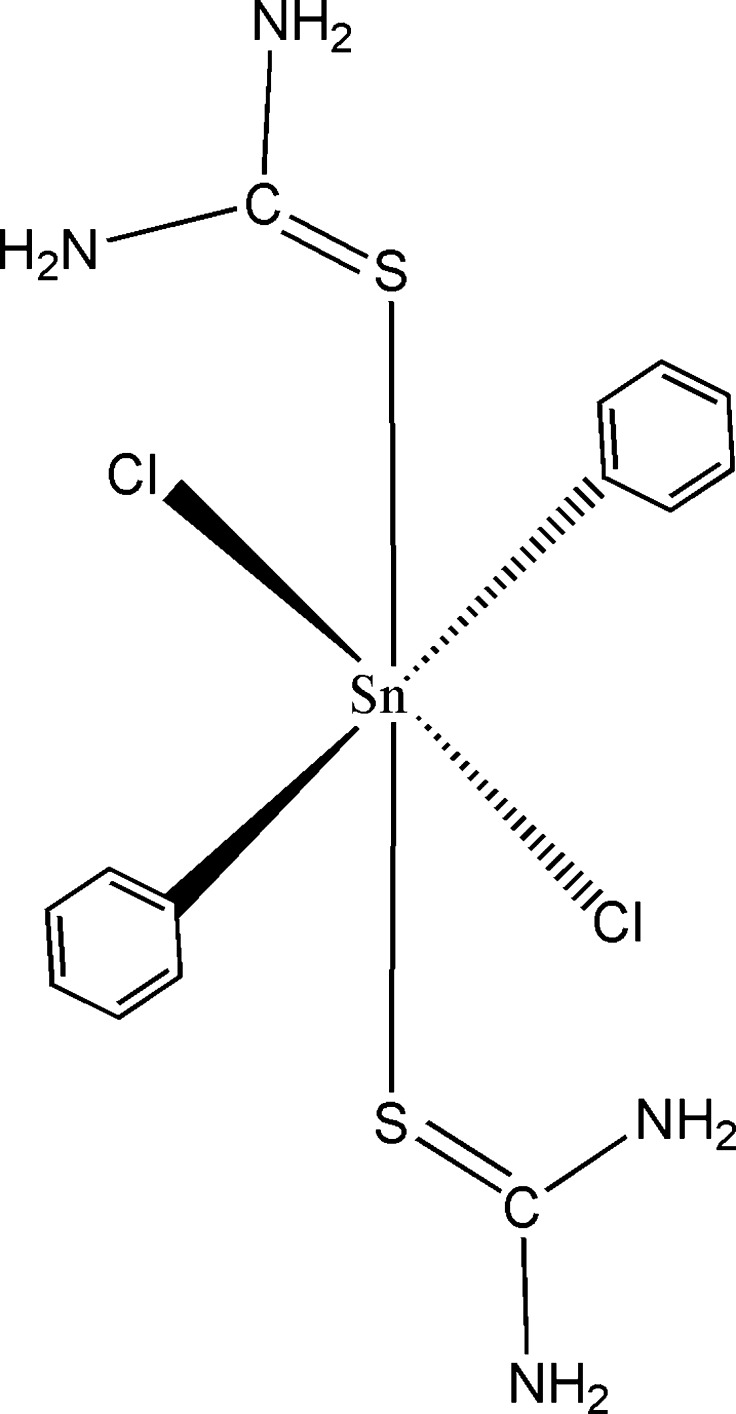


## Experimental

### 

#### Crystal data

[Sn(C_6_H_5_)_2_Cl_2_(CH_4_N_2_S)_2_]
*M* *_r_* = 496.03
Tetragonal, 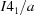

*a* = 14.6401 (2) Å
*c* = 17.7899 (3) Å
*V* = 3812.95 (10) Å^3^
*Z* = 8
Mo *K*α radiation
μ = 1.84 mm^−1^
*T* = 150 K
0.35 × 0.35 × 0.25 mm

#### Data collection

Nonius KappaCCD diffractometer
Absorption correction: multi-scan (*SORTAV*; Blessing, 1995 ▶) *T*_min_ = 0.565, *T*_max_ = 0.656
23870 measured reflections
2183 independent reflections
1892 reflections with *I* > 2σ(*I*)
*R*_int_ = 0.041

#### Refinement

*R*[*F* ^2^ > 2σ(*F* ^2^)] = 0.019
*wR*(*F* ^2^) = 0.051
*S* = 1.06
2183 reflections
123 parameters
H atoms treated by a mixture of independent and constrained refinement
Δρ_max_ = 0.40 e Å^−3^
Δρ_min_ = −0.72 e Å^−3^

### 

Data collection: *COLLECT* (Nonius, 1999 ▶); cell refinement: *DENZO* and *SCALEPACK* (Otwinowski & Minor, 1997 ▶); data reduction: *DENZO* and *SCALEPACK*; program(s) used to solve structure: *SIR97* (Altomare *et al.*, 1999 ▶); program(s) used to refine structure: *SHELXL97* (Sheldrick, 2008 ▶); molecular graphics: *ORTEP-3 for Windows* (Farrugia, 2012 ▶); software used to prepare material for publication: *WinGX* (Farrugia, 2012 ▶).

## Supplementary Material

Crystal structure: contains datablock(s) I, New_Global_Publ_Block. DOI: 10.1107/S1600536813024343/wm2764sup1.cif

Structure factors: contains datablock(s) I. DOI: 10.1107/S1600536813024343/wm2764Isup2.hkl

Additional supplementary materials:  crystallographic information; 3D view; checkCIF report

## Figures and Tables

**Table 1:** Hydrogen-bond geometry (Å, °)

*D*—H⋯*A*	*D*—H	H⋯*A*	*D*⋯*A*	*D*—H⋯*A*
N1—H1*B*⋯Cl2	0.86 (3)	2.50 (3)	3.345 (2)	166 (2)
N2—H2*A*⋯Cl1^i^	0.81 (3)	2.41 (3)	3.2119 (19)	170 (3)

Symmetry code: (i) 
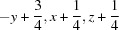
.

**Fig. 1.**
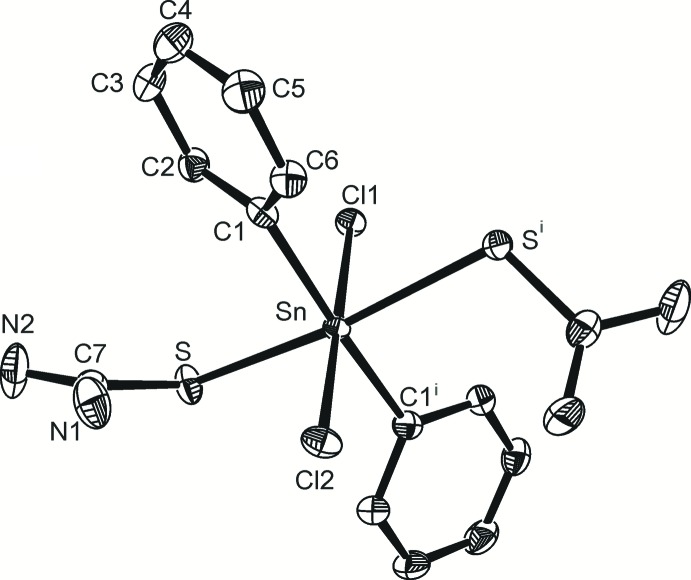
The molecular structure of (I) showing the atomic labelling scheme. Displacement ellipsoids are drawn at the 50% probability level; H atoms were omitted for clarity. [Symmetry code i) -x, 1/2 - y, z.]

**Fig. 2.**
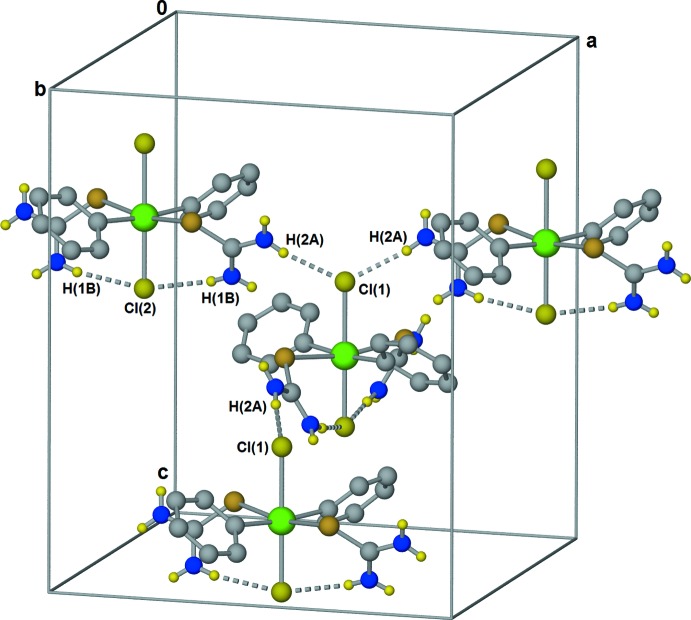
A section of the lattice structure of (I) showing both the intermolecular N(2)—H(2 A)···Cl(1) and intramolecular N(1)—H(1B)···Cl(2) hydrogen bonding interactions as dashed lines. Only selected hydrogen atoms have been included for clarity.

## supplementary crystallographic information

### 1. Comment

Many Sn(C_6_H_5_)_2_Cl_2_ adducts have previously been reported and structurally characterized (Kapoor *et al.*, 2005; Müller *et al.*, 2008; Sadiq-ur-Rehman *et al.*, 2007; Sow *et al.*, 2012; Zhang *et al.*, 2006). Organotin (IV) compounds exhibiting biological (e.g antitumour) activity have also been reported (Nath *et al.*, 2001; Pellerito & Nagy, 2002). On allowing Sn(C_6_H_5_)_2_Cl_2_ and SC(NH_2_)_2_ to react, crystals of the title compound, [Sn(C_6_H_5_)_2_Cl_2_(CH_4_N_2_S)_2_], (I), were obtained and the structure determined.

The molecule in the structure of compound (I) is located on a twofold rotation axis. The Sn^IV^ atom has an octahedral *trans, trans, trans*-C_2_S_2_Cl_2_ coordination sphere (Fig. 1), defined by two carbon atoms of symmetry-related phenyl groups [2.1622 (17) Å], two Cl [2.5194 (6), 2.6224 (6) Å] and two sulfur atoms of the thiourea ligands [2.6755 (4) Å]. The Sn—S bond is somewhat shorter than the analogous bond [2.6945 (7) Å] in the related structure of [Sn_2_(C_2_O_4_)(C_6_H_5_)_6_(CH_4_N_2_S)_2_] (Sow *et al.*, 2012), but is in the range of other Sn—S bonds reported for tin(IV) structures containing thiourea ligands (Donaldson *et al.*, 1984; Wirth *et al.*, 1998).

The Sn—Cl2 distance [2.5194 (6) Å] is shorter than the Sn—Cl1 distance [2.6224 (6) Å] as a consequence of the involvement of Cl1 in strong intermolecular N2—H2A···Cl1 hydrogen bond formation (Table 1), which leads to the formation of a three-dimensional network structure. Somewhat weaker intramolecular N1—H1B···Cl2 hydrogen bonds are also present (Fig. 2). When compared to the unique Sn—Cl distance in {[((CH_3_)_3_C)_2_(CH_3_)PO]_2_^.^Sn(C_6_H_5_)_2_Cl_2_} [2.5567 (16) Å] (Müller *et al.*, 2008) or the two Sn—Cl distances in {18-crown-6^.^Sn(C_6_H_5_)_2_Cl_2_^.^2H_2_O} [2.5521 (7), 2.5627 (7) Å] (Amini *et al.*, 2002), two adducts in which the Cl atoms are not involved in hydrogen bonding, it appears that in (I) the intermolecular hydrogen bonding has led to a weakened Sn—Cl1 bond and a concomitant strengthening of Sn—Cl2. Moreover, the strength of Sn—Cl2 in comparison with examples of other Sn—Cl bonds where the halogen is not involved in hydrogen bonding suggests that the intramolecular hydrogen bonds are weaker than suggested by their interatomic separation.

### 2. Experimental

All chemicals were purchased from Aldrich (Germany) and used without any further purification. The title compound, [Sn(C_6_H_5_)_2_Cl_2_(CH_4_N_2_S)_2_], has been synthesized from the reaction between Sn(C_6_H_5_)_2_Cl_2_ (0.250 g, 0.727 mmol) and SC(NH_2_)_2_ (0.111 g, 1.454 mmol) in a 1:2 ratio in absolute ethanol solution. After stirring for two hours, a clear solution was obtained that was slowly evaporated at room temperature yielding colourless crystals (weight, yield 69%) with a melting point of 515 K. Analytical data for C_14_H_18_Cl_2_N_4_S_2_Sn (found) %C: 33.90 (33.87); %H: 3.66 (3.63); %N: 11.29(11.27); %S: 12.93 (12.90); %Sn: 23.93 (23.98).

### 3. Refinement

Hydrogen atoms bonded to the N atom have been located in difference Fourier maps and have been freely refined. The other hydrogen atoms have been placed onto calculated position and refined using a riding model, with C—H distances of 0.95 Å and *U*_iso_(H)= 1.2*U*_eq_(C).

### Crystal data

**Table:**

[Sn(C_6_H_5_)_2_Cl_2_(CH_4_N_2_S)_2_]	*D*_x_ = 1.728 Mg m^−^^3^
*M**_r_* = 496.03	Mo *K*α radiation, λ = 0.71073 Å
Tetragonal, *I*4_1_/*a*	Cell parameters from 13789 reflections
Hall symbol: -I 4ad	θ = 2.9–27.5°
*a* = 14.6401 (2) Å	µ = 1.84 mm^−^^1^
*c* = 17.7899 (3) Å	*T* = 150 K
*V* = 3812.95 (10) Å^3^	Block, colourless
*Z* = 8	0.35 × 0.35 × 0.25 mm
*F*(000) = 1968	

### Data collection

**Table:**

Nonius KappaCCD diffractometer	2183 independent reflections
Radiation source: fine-focus sealed tube	1892 reflections with *I* > 2σ(*I*)
Graphite monochromator	*R*_int_ = 0.041
152 2.0 degree images with ω scans	θ_max_ = 27.5°, θ_min_ = 3.3°
Absorption correction: multi-scan (*SORTAV*; Blessing, 1995)	*h* = −18→18
*T*_min_ = 0.565, *T*_max_ = 0.656	*k* = −18→18
23870 measured reflections	*l* = −23→23

### Refinement

**Table:**

Refinement on *F*^2^	Secondary atom site location: difference Fourier map
Least-squares matrix: full	Hydrogen site location: inferred from neighbouring sites
*R*[*F*^2^ > 2σ(*F*^2^)] = 0.019	H atoms treated by a mixture of independent and constrained refinement
*wR*(*F*^2^) = 0.051	*w* = 1/[σ^2^(*F*_o_^2^) + (0.0219*P*)^2^ + 4.1509*P*] where *P* = (*F*_o_^2^ + 2*F*_c_^2^)/3
*S* = 1.06	(Δ/σ)_max_ = 0.001
2183 reflections	Δρ_max_ = 0.40 e Å^−^^3^
123 parameters	Δρ_min_ = −0.72 e Å^−^^3^
0 restraints	Extinction correction: *SHELXL97* (Sheldrick, 2008), Fc^*^=kFc[1+0.001xFc^2^λ^3^/sin(2θ)]^-1/4^
Primary atom site location: structure-invariant direct methods	Extinction coefficient: 0.00076 (6)

### Special details

**Table:**

Geometry. All e.s.d.'s (except the e.s.d. in the dihedral angle between two l.s. planes) are estimated using the full covariance matrix. The cell e.s.d.'s are taken into account individually in the estimation of e.s.d.'s in distances, angles and torsion angles; correlations between e.s.d.'s in cell parameters are only used when they are defined by crystal symmetry. An approximate (isotropic) treatment of cell e.s.d.'s is used for estimating e.s.d.'s involving l.s. planes.
Refinement. Refinement of *F*^2^ against ALL reflections. The weighted *R*-factor *wR* and goodness of fit *S* are based on *F*^2^, conventional *R*-factors *R* are based on *F*, with *F* set to zero for negative *F*^2^. The threshold expression of *F*^2^ > σ(*F*^2^) is used only for calculating *R*-factors(gt) *etc*. and is not relevant to the choice of reflections for refinement. *R*-factors based on *F*^2^ are statistically about twice as large as those based on *F*, and *R*- factors based on ALL data will be even larger.

### Fractional atomic coordinates and isotropic or equivalent isotropic displacement parameters (Å^2^)

**Table:**

	*x*	*y*	*z*	*U*_iso_*/*U*_eq_	
Sn	0.0000	0.2500	0.371721 (7)	0.01479 (8)	
Cl1	0.0000	0.2500	0.22431 (3)	0.01928 (13)	
Cl2	0.0000	0.2500	0.51334 (3)	0.02256 (13)	
S	0.15011 (3)	0.35281 (3)	0.35755 (3)	0.02464 (11)	
N1	0.22306 (14)	0.26557 (13)	0.47461 (10)	0.0378 (4)	
H1A	0.2663 (18)	0.2448 (17)	0.4972 (14)	0.054 (8)*	
H1B	0.1685 (19)	0.2538 (17)	0.4898 (14)	0.052 (7)*	
N2	0.32157 (12)	0.32246 (17)	0.38782 (12)	0.0463 (5)	
H2A	0.3644 (18)	0.3077 (18)	0.4143 (15)	0.056 (7)*	
H2B	0.331 (2)	0.348 (2)	0.3459 (19)	0.076 (10)*	
C1	0.07672 (12)	0.12407 (11)	0.36491 (8)	0.0191 (3)	
C2	0.15058 (11)	0.11573 (11)	0.31618 (9)	0.0220 (3)	
H2	0.1712	0.1675	0.2888	0.026*	
C3	0.19442 (12)	0.03197 (13)	0.30729 (10)	0.0287 (4)	
H3	0.2448	0.0266	0.2739	0.034*	
C4	0.16417 (13)	−0.04356 (13)	0.34738 (11)	0.0325 (4)	
H4	0.1930	−0.1011	0.3406	0.039*	
C5	0.09216 (13)	−0.03516 (12)	0.39710 (11)	0.0303 (4)	
H5	0.0724	−0.0867	0.4252	0.036*	
C6	0.04852 (11)	0.04849 (11)	0.40608 (9)	0.0231 (3)	
H6	−0.0008	0.0540	0.4405	0.028*	
C7	0.23733 (12)	0.30925 (11)	0.41088 (10)	0.0247 (4)	

### Atomic displacement parameters (Å^2^)

**Table:**

	*U*^11^	*U*^22^	*U*^33^	*U*^12^	*U*^13^	*U*^23^
Sn	0.01624 (10)	0.01267 (10)	0.01545 (10)	−0.00079 (5)	0.000	0.000
Cl1	0.0222 (3)	0.0212 (3)	0.0144 (2)	−0.00041 (19)	0.000	0.000
Cl2	0.0323 (3)	0.0204 (3)	0.0150 (3)	−0.0046 (2)	0.000	0.000
S	0.0184 (2)	0.0218 (2)	0.0337 (2)	−0.00452 (16)	−0.00682 (17)	0.00877 (17)
N1	0.0324 (10)	0.0431 (10)	0.0380 (9)	−0.0012 (8)	−0.0151 (8)	0.0110 (8)
N2	0.0187 (9)	0.0828 (16)	0.0373 (10)	0.0101 (9)	−0.0039 (8)	0.0048 (10)
C1	0.0211 (8)	0.0180 (8)	0.0183 (7)	−0.0031 (6)	−0.0033 (6)	−0.0004 (6)
C2	0.0198 (8)	0.0217 (8)	0.0246 (8)	−0.0008 (6)	0.0003 (6)	0.0015 (6)
C3	0.0201 (9)	0.0310 (10)	0.0352 (10)	0.0061 (7)	0.0019 (7)	−0.0032 (7)
C4	0.0295 (10)	0.0244 (9)	0.0437 (11)	0.0092 (7)	−0.0056 (8)	0.0005 (8)
C5	0.0331 (10)	0.0210 (9)	0.0368 (9)	0.0020 (7)	−0.0030 (8)	0.0084 (7)
C6	0.0246 (8)	0.0216 (8)	0.0231 (8)	0.0003 (6)	−0.0003 (6)	0.0036 (6)
C7	0.0238 (8)	0.0231 (8)	0.0271 (9)	0.0021 (6)	−0.0058 (7)	−0.0057 (7)

### Geometric parameters (Å, º)

**Table:**

Sn—C1	2.1622 (17)	N2—H2B	0.85 (3)
Sn—C1^i^	2.1622 (17)	C1—C6	1.390 (2)
Sn—Cl2	2.5194 (6)	C1—C2	1.391 (2)
Sn—Cl1	2.6224 (6)	C2—C3	1.393 (2)
Sn—S^i^	2.6755 (4)	C2—H2	0.9500
Sn—S	2.6755 (4)	C3—C4	1.388 (3)
S—C7	1.7137 (17)	C3—H3	0.9500
N1—C7	1.318 (2)	C4—C5	1.382 (3)
N1—H1A	0.81 (3)	C4—H4	0.9500
N1—H1B	0.86 (3)	C5—C6	1.391 (2)
N2—C7	1.314 (2)	C5—H5	0.9500
N2—H2A	0.81 (3)	C6—H6	0.9500
			
C1—Sn—C1^i^	173.57 (8)	C6—C1—C2	119.30 (16)
C1—Sn—Cl2	93.21 (4)	C6—C1—Sn	119.67 (12)
C1^i^—Sn—Cl2	93.21 (4)	C2—C1—Sn	120.90 (12)
C1—Sn—Cl1	86.79 (4)	C1—C2—C3	120.40 (16)
C1^i^—Sn—Cl1	86.79 (4)	C1—C2—H2	119.8
Cl2—Sn—Cl1	180.0	C3—C2—H2	119.8
C1—Sn—S^i^	86.66 (4)	C4—C3—C2	119.72 (16)
C1^i^—Sn—S^i^	92.73 (4)	C4—C3—H3	120.1
Cl2—Sn—S^i^	95.405 (10)	C2—C3—H3	120.1
Cl1—Sn—S^i^	84.595 (10)	C5—C4—C3	120.09 (17)
C1—Sn—S	92.73 (4)	C5—C4—H4	120.0
C1^i^—Sn—S	86.66 (4)	C3—C4—H4	120.0
Cl2—Sn—S	95.405 (10)	C4—C5—C6	120.17 (16)
Cl1—Sn—S	84.595 (10)	C4—C5—H5	119.9
S^i^—Sn—S	169.19 (2)	C6—C5—H5	119.9
C7—S—Sn	110.52 (6)	C1—C6—C5	120.28 (16)
C7—N1—H1A	119.0 (18)	C1—C6—H6	119.9
C7—N1—H1B	120.9 (17)	C5—C6—H6	119.9
H1A—N1—H1B	120 (2)	N2—C7—N1	119.24 (18)
C7—N2—H2A	120.3 (19)	N2—C7—S	118.15 (15)
C7—N2—H2B	119 (2)	N1—C7—S	122.58 (15)
H2A—N2—H2B	120 (3)		

Symmetry code: (i) −*x*, −*y*+1/2, *z*.

### Hydrogen-bond geometry (Å, º)

**Table:**

*D*—H···*A*	*D*—H	H···*A*	*D*···*A*	*D*—H···*A*
N1—H1*B*···Cl2	0.86 (3)	2.50 (3)	3.345 (2)	166 (2)
N2—H2*A*···Cl1^ii^	0.81 (3)	2.41 (3)	3.2119 (19)	170 (3)

Symmetry code: (ii) −*y*+3/4, *x*+1/4, *z*+1/4.
